# Editorial: Early and accurate diagnosis and regulatory mechanism of lymph node metastasis in head and neck carcinoma

**DOI:** 10.3389/fonc.2024.1389158

**Published:** 2024-04-09

**Authors:** Fa-yu Liu, Keith L. Kirkwood, Zhien Feng

**Affiliations:** ^1^ Department of Oral Maxillofacial-Head and Neck Surgery, School and Hospital of Stomatology, China Medical University, Liaoning Provincial Key Laboratory of Oral Diseases, Shenyang, Liaoning, China; ^2^ Department of Oral Biology, School of Dental Medicine, University at Buffalo, Buffalo, NY, United States; ^3^ Department of Head & Neck/Plastic and Reconstructive Surgery, Roswell Park Comprehensive Cancer Center, Buffalo, NY, United States; ^4^ Department of Oral and Maxillofacial-Head and Neck Oncology, Beijing Stomatological Hospital, Capital Medical University, Beijing, China

**Keywords:** head and neck carcinoma, lymph node metastasis (LNM), early and accurate diagnosis, radiomics, deep learning

Head and neck carcinoma is the sixth most common cancer worldwide, with malignant tumors on four main anatomical sites: the oral cavity, nasal cavity, pharynx and larynx. Sometimes researchers also include thyroid cancer in head and neck cancer considering its location and similar features. Head and neck cancers tend to develop local lymph node metastasis (LNM). Although, following conventional surgery, radiotherapy, chemotherapy and other treatments, patients with LNM have higher tumor recurrence and lower disease-specific survival than without LNM, suggesting the adoption of more radical therapy to head and neck cancer with LNM (1). Therefore, early and accurate diagnosis of LNM is critical for proper treatment planning.

Generally, head and neck surgeons use ultrasound (US), magnetic resonance imaging (MRI), computed tomography (CT) and Positron Emission Tomography (PET-CT) to identify tumor size and LNM. However, these monitoring techniques heavily rely on physician judgment, which is inconsistent, resulting in their unsuitability for population-wide screening (2). Furthermore, some small or occult (early stage) LNM shows different or missed diagnoses (3, 4). Radiomics and deep learning, which are branches of artificial intelligence, provide high-dimensional radiomic features extracted from medical images in a non-invasive way and build disease classification models (5). In this Research Topic on early and accurate diagnosis and regulatory mechanisms of lymph node metastasis in head and neck carcinoma, six articles were selected, including four articles on radiomics and deep learning, suggesting that they are current research hotspots in this field. It is interesting that these four articles are all based on US results on thyroid cancer. This may be due to the high morbidity of thyroid cancer and the universal use of US examinations, which feed into the use of machine learning analyses on robust datasets. In the other two articles, one paper focuses on the relationship between lymph node metastasis and distant metastasis-free survival in parotid gland cancer, and the other paper addresses how to distinguish lymph node false negatives in head and neck squamous cell carcinoma patients using FDG-PET/CT.

Liu et al. conducted a retrospective study of 541 patients with papillary thyroid carcinoma whose clinical lymph nodes were diagnosed as negative before surgery. Here, the authors compared the clinical data, conventional US features, and US elastography indices between the lymph node-positive group (demonstrated by postoperative pathological examination) and the lymph node-negative group, and found that age <34 years, male sex, strain rate ratio (SRR) >2 and tumor size >9.95 mm were independent predictors of occult clinical cervical LNM. These results may help clinicians choose appropriate treatment strategies despite the obvious study limitation where the area under the curve (AUC) of different predictive factors is below 0.7, indicating low accuracy.

To explore the efficiency and potential clinical application value of predicting cervical lymph node metastasis in papillary thyroid carcinoma, Zhang et al. developed an easy-to-use and non-invasive US radiology nomogram. The authors initially screened thyroglobulin (TG) level, tumor size, aspect ratio and radiomic signatures as independent factors for predicting large numbers of cervical LNM before surgery, then combined them together and constructed a US radiomic nomogram. This was found to have good predictive ability, with an AUC of 0.935 in the training set and 0.782 in the testing set. 

In the field of deep learning, Zhao et al. retrieved ultrasound reports for 3059 cervical lymph nodes (CLN) from 2398 patients. All CLNs were confirmed by a fine needle aspiration biopsy. The authors used the Y-Net network model to segment and differentiate lymph nodes, and found that the Y-Net model yielded better accuracy than the original ultrasound reports in differentiating LNM. This article highlights a robust CLN sample size, suggesting credible findings. Furthermore, Dai et al. collected 498 cases of unifocal papillary thyroid carcinoma, acquired grayscale ultrasound, color Doppler flow imaging (CDFI) and elastography images, and compared unimodal and multimodal analyses by these three types of classifier models. The results showed that the multimodal SVM model achieved the best predictive value compared to other models. This article has three highlights: 1) they compared unimodal and multimodal, 2) they specifically studied the performance of cN0 patients, which is more valuable in clinical work, and 3) they constructed an independent testing set (validation set), which confirmed their experimental data sets.

Squamous cell carcinoma (SCC) is the most common type of head and neck cancer. FDG-PET/CT is widely used to detect lymph node metastasis in head and neck SCC, but it has a significant false negative rate. In order to find the cause of this phenomenon, Meng et al. detected the markers of glucose metabolism, amino acid metabolism, and lipid metabolism in 92 primary cases of HNSCC with FDG-PET/CT examination, and found that CD36 expression levels in primary lesions could be a promising biomarker for distinguishing false negative nodes. The authors also demonstrated a pro-invasive biological effect of CD36 in an *in vitro* experiment. The results appear to be a significant adjunct to the FDG-PET/CT diagnosis.

Parotid gland cancer is relatively uncommon, accounting for less than 3% of all head and neck cancers. Parotid cancers with distant metastases are even rarer, contributing to the paucity of literature on factors influencing distant metastasis-free survival (DMFS). Meng et al. retrospectively collected 490 surgically treated parotid cancers in 27 years, and analyzed the influence of the number of positive lymph nodes (LNs) and extranodal extension (ENE) on the distant metastasis of parotid carcinoma. The results showed that parotid lymph node metastasis was related to a decrease in DMFS, and this effect may be driven by the number of positive LNs, rather than ENE. This is the first article to systematically describe the relationship between LNM and DMFS with such a large number of specimens.

This Research Topic summarizes retrospective research on the advanced diagnostic method of lymph node metastasis of head and neck cancer, emphasizes the application of radiomics and deep learning systems, raises the potential biomarker CD36 to provide more personalized treatment for patients, and introduces the relationship between LNM and DMFS in parotid gland cancer. In conclusion, the development of research methods based on medical images, such as radiomics and artificial neural networks, is a new trend that can provide non-invasive, rapid and accurate diagnostic means, helping physicians better understand the characteristics of disease progression, thus improving the accuracy of treatment decisions. At the same time, by analyzing objective indicators, various imaging features can be linked with clinical prognostic factors, which have broad application prospects and are playing an increasingly important role in clinical diagnosis and treatment.

## Author contributions

FL: Funding acquisition, Writing – original draft. KK: Conceptualization, Writing – review & editing. ZF: Writing – original draft, Writing – review & editing.
